# High CD133 Expression in the Nucleus and Cytoplasm Predicts Poor Prognosis in Non-Small Cell Lung Cancer

**DOI:** 10.1155/2015/986095

**Published:** 2015-01-18

**Authors:** Minjie Huang, Huijun Zhu, Jian Feng, Songshi Ni, Jianfei Huang

**Affiliations:** ^1^Department of Respiratory Medicine, Affiliated Hospital of Nantong University, Nantong, Jiangsu 226001, China; ^2^Department of Pathology, Affiliated Hospital of Nantong University, Nantong, Jiangsu 226001, China

## Abstract

*Objective*. The aim of this study was to investigate the expression of Prominin-1 (CD133) in cancer cells and its potential value as a prognostic indicator of survival in patients with non-small cell lung cancer (NSCLC). *Methods*. Cancerous tissues and matched normal tissues adjacent to the carcinoma from 239 NSCLC patients were obtained immediately after surgery. Immunohistochemistry of tissue microarrays was used to characterize the expression of CD133 in NSCLC and adjacent tissues. The correlation of CD133 expression with clinical characteristics and prognosis was determined by statistical analysis. *Results*. CD133 protein expression levels in both the cytoplasm and nucleus were significantly higher in NSCLC tissues compared with corresponding peritumoral tissue (*P* < 0.05). CD133 expression in the nucleus of NSCLC cells was related to tumor diameter (*P* = 0.027), tumor differentiation (*P* < 0.001), and TNM stage (*P* = 0.007). Kaplan-Meier survival and Cox regression analyses revealed that high CD133 expression in the nucleus was an independent predictor of poor prognosis of NSCLC, as was high cytoplasmic CD133 expression (*P* < 0.001). *Conclusion*. Our findings provide the first evidence that high expression of CD133 in both the nucleus and cytoplasm is associated with poor prognosis in NSCLC.

## 1. Introduction

Lung cancer is a leading cause of cancer death worldwide [1, 2]. Non-small cell lung cancers (NSCLCs) have a high morbidity and mortality rate and account for 80–85% of all lung cancers, which include adenocarcinomas, squamous cell carcinomas of the lung, and large cell undifferentiated carcinomas [2]. Surgical removal remains the best curative option for patients with early-stage NSCLC. However, despite recent advances in cancer treatment, the prognosis for those with NSCLC remains poor, with a 5-year overall survival rate of less than 15% [3, 4].

Much work towards molecular-targeted therapy for lung cancer has occurred in recent years. The identification of genetic alterations that drive cancer progression and the application of targeted therapy have prolonged survival of NSCLC patients [5].

Research into new prognostic markers is important to establish adequate therapeutic strategies for lung cancer. Several recent investigations have demonstrated the presence of cancer stem cells (CSCs) in tumor specimens, and the detection of stem cell markers (CD24−/CD44+, CD133, and ALDHA1) can provide useful prognostic information [6, 7].

Hamburger and Salmon [8] first proposed the CSC theory, which posits that cancer originates from uncommon cells that demonstrate pluripotency and selfrenewal [9]. These CSCs are responsible for the initiation, progression, and recurrence of cancer. Therefore, CSC markers are useful in the identification and study of CSCs and their role in the cause of tumorigenesis. A variety of molecules have been investigated as putative markers of CSCs in malignancies, with CD133 being one of the most commonly used markers.

A growing number of CD133-positive cancer cells have been identified in lung cancer [10, 11]. Eramo et al. [12] observed that a rare population of CD133-positive CSC-like cells were able to self-renew and generate an unlimited progeny of nontumorigenic cells, whereas CD133-negative cancer cells lacked this potential. However, the association between the location of CD133 expression and the clinicopathological characteristics, clinical features, and survival outcome in lung cancer remains unknown [13].

A meta-analysis [13] has suggested that CD133 expression is associated with poor tumor differentiation and lymph node metastasis. Poor differentiation and metastasis were also significantly associated with poor survival of cancer. Therefore, positive CD133 expression was most likely correlated with poor prognosis of lung cancer.

To date, few findings have been published regarding changes in the subcellular localization of these markers. Although a lot of papers have reported on the distribution of cell-surface proteins in the tumor cell cytoplasm in association with membrane localization [12, 14–16], they have ignored the nuclear expression of CD133 and its nuclear sublocalization has been described only in few papers [17, 18].

In this study, we explore the nuclear localization of CD133 in human NSCLC tumors compared with adjacent tissues and examine the correlation between nuclear localization of CD133 and patient prognosis. We demonstrate the prognostic significance of the nuclear localization of CD133 expression in NSCLC and present the potential value of this marker as a prognostic indicator of survival in patients with NSCLC.

## 2. Materials and Methods

### 2.1. NSCLC Patient Specimens

Formalin-fixed, paraffin-embedded tumor samples from 239 NSCLC cases and 123 matched peritumoral tissue specimens were collected in NSCLC patients from the Department of Pathology, the Affiliated Hospital of Nantong University from 2004 to 2009. All cases were reevaluated for grade and histological type by two independent pathologists. The mean age of patients at the time of surgery was 63 years (range of 35–83 years). Other original clinical data were also collected, including gender, age, tumor size, histological type, tumor differentiation, TNM stage, and 5-year follow-up survival. No patients had received chemotherapy or radiation before the operation. Tumor staging was performed in accordance with the guidelines of the 7th edition of TNM staging in lung cancer [19]. Written informed consent and any related picture were acquired from each patient for publication of this study. This research was approved by the local Human Research Ethics Committee of the Affiliated Hospital of Nantong University, Nantong, China.

### 2.2. Tissue Microarray (TMA) Construction and Immunohistochemistry (IHC) Analysis

The 239 NSCLCs and 123 normal tumor-adjacent tissues were prepared and used for this present study. We used a Tissue Microarray System (Quick-Ray, UT06, UNITMA, Korea) in the Department of Clinical Pathology, Nantong University Hospital, Jiangsu, China, to produce 2 mm thick paraffin-embedded NSCLC TMA sections. Core tissue biopsies (2 mm in diameter) were taken from individual paraffin-embedded sections and arranged in the new recipient paraffin blocks. TMA blocks were cut into 4 *μ*m sections and placed on super frost-charged glass microscope slides.

IHC analysis was performed as previously described [20]. Deparaffinized sections (4 *μ*m thick) from array blocks were separately stained on an Autostainer Universal Staining System (LabVision, Kalamazoo, MI, USA) using polyclonal rabbit anti-CD133 antibody (LifeSpan BioSciences Inc., Seattle, WA, USA). The secondary antibody used was horseradish peroxidase-conjugated anti-rabbit antibody (Dako Cytomation, Carpinteria, CA, USA). For negative controls, phosphate-buffered saline was used instead of the primary antibody. Blind CD133 immunostaining evaluation and independent observation were simultaneously performed. IHC results were analyzed according to a previously described method [21]. Staining intensity was scored as follows: 0 (negative), 1 (weakly positive), 2 (moderately positive), and 3 (strongly positive). The percentage of CD133-positive cells was also scored into four categories, with a score of 1 given for <10%, 2 for 11–50%, 3 for 51–80%, and 4 for >81%. The product of the intensity and percentage scores was used as the final CD133 staining score. The cutoff point for the CD133 expression score that was statistically significant in terms of survival was set using the X-tile software program (The Rimm Lab at Yale University; http://www.tissuearray.org/rimmlab/) as described previously [21]. The degree of CD133 staining was quantified using a two-level grading system, and staining scores were defined as follows: 0-1, low expression, and 2–9, high expression.

### 2.3. Statistical Analysis

Associations between CD133 expression and categorical variables were analyzed using chi-square tests or Fisher exact tests, as appropriate. Survival curves were estimated using the Kaplan-Meier method, and differences in survival distributions were evaluated using the log-rank test. Univariate analysis using Cox proportional hazards modeling was applied to determine correlations between various factors and overall survival. The level of significance was set at *P* < .05. Statistical analyses were performed using STATA 9.0 software (Stata Corporation, College Station, TX, USA).

## 3. Results

### 3.1. Expression of CD133 Protein in NSCLC and Peritumoral Tissues

**Figure 1:**
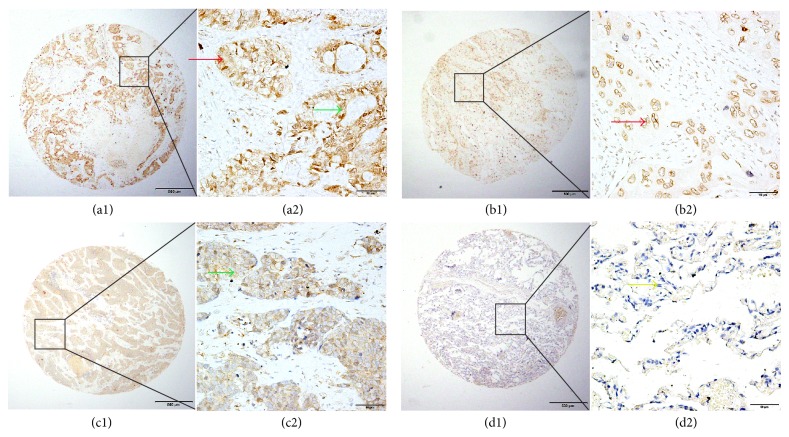
Representative patterns of CD133 protein expression in NSCLC and normal lung tissue. (a1) and (a2) Adenocarcinoma tissue and adjacent normal lung tissue. The expression of CD133 in tumor tissue was higher than that in adjacent normal lung tissue by IHC staining. The red arrow indicates positive CD133 protein expression in the nucleus of adenocarcinoma cells. The green arrow indicates strong CD133 staining in the cytoplasm of tumor cells. (b1) and (b2) Squamous cell carcinoma of the lung. The expression of CD133 protein was positive in lung carcinoma tissues. The red arrow indicates positive CD133 protein expression in the nucleus of tumor cells. (c1) and (c2) Squamous cell carcinoma of the lung. The green arrow indicates CD133 staining in the cytoplasm of tumor cells. (d1) and (d2) Normal lung tissue. The expression of CD133 protein was negative in both the cytoplasm and nucleus. The yellow arrow indicates that expression of CD133 in alveolar epithelial cells is negative.

To further investigate the expression of CD133 protein in carcinomas and the corresponding adjacent tissues, we performed IHC analysis on primary patient NSCLC specimens. CD133 was detected at various levels, primarily in the nucleus and cytoplasm of cells (Figure 1). High CD133 expression was detected in 57.30% (137/239) of NSCLC samples, compared with 26.02% (32/123) of adjacent matched tumor tissues. The typically observed CD133 staining patterns are shown in Figure 1.

### 3.2. Association between CD133 Expression and Clinicopathological Parameters of NSCLC

The association between high CD133 expression and the selected clinicopathological variables in NSCLC patients is shown in Table 1. High CD133 expression in the nucleus was associated with tumor diameter (*P* = .027), tumor differentiation (*P* < .001), and TNM stage (*P* = .007). No significant association between CD133 expression and other clinical parameters such as gender, age, and histological type was identified (Table 1). High CD133 expression in the cytoplasm was also associated with tumor diameter (*P* = .022), tumor differentiation (*P* < .001), and TNM stage (*P* = .008), while similarly no significant association between CD133 expression and other clinical parameters was identified (Table 1).

When CD133 expression was low or absent in both the cytoplasm and nucleus (92/239 samples), the average survival time was 57.46 years. In contrast, when both cytoplasmic and nuclear CD133 expression were high (115/239 samples), the average survival time was 17.45 years. Furthermore, when cytoplasmic CD133 expression was low but nuclear expression was high (22/239 samples), the average survival time was 38.82 years while when nuclear CD133 expression was low but cytoplasmic expression was high (10/239), the average survival time was 49.10 years.

### 3.3. Survival Analysis

**Figure 2:**
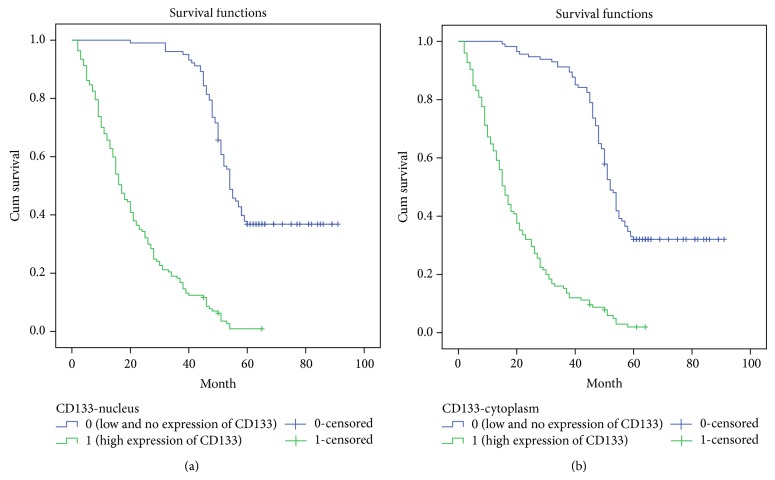
Kaplan-Meier survival curves following surgical therapy in NSCLC. (a) Patients with high CD133 expression in the nucleus of tumor cells (green line) exhibited significantly poorer survival compared with the low or no expression group (blue line). (b) Patients with high CD133 expression in the cytoplasm of tumor cells (green line) exhibited significantly poorer survival compared with the low or no expression group (blue line).

Based on univariate Cox regression analyses for all factors, high CD133 expression in both the cytoplasm and nucleus was a significant (*P* < .001) prognostic factor for NSCLC (Table 2). Tumor differentiation (*P* = .001) and tumor diameter (*P* = .035) were also closely related to patient survival. The multivariate Cox regression model further demonstrated that CD133 expression (*P* < .001), tumor diameter (*P* = .005), and tumor differentiation (*P* = .015) were the strongest predictors of patient survival (Table 2). Kaplan-Meier survival curves showed that NSCLC patients with low and no CD133 expression had a significantly favorable survival time (Figure 2).

## 4. Discussion

Lung cancer is the most preventable cancer, but once established its prognosis is poor. The 5-year survival rate is low because of late presentation, disease relapse, and a low rate of curative therapy [22]. Understanding lung cancer pathogenesis may improve future human therapies, and preliminary evidence has pointed to the existence of cancer stem cells (CSCs) in lung cancer [22].

CD133, also known as Prominin-1, is a member of the pentaspan transmembrane (5-TM) glycoprotein family. In humans, the Prominin-1 gene is located on chromosome 4p15 and encodes a 120-kD transmembrane glycoprotein [23], which localizes to membrane protrusions. CD133 is widely used to identify and isolate stem cells and CSCs. It was first described as a hematopoietic stem cell marker and later found on certain types of leukemic cells [23]; however, its precise function remains unclear. It is hypothesized to be associated with cell-cell interactions or signal transduction [24]. Recently, expression of CD133 in CSCs from a variety of solid tumors has been reported, including tumors from the brain [25], liver [26], ovary [27], colon [28], lung [29], and endometrium [30].

In 2009, Tirino et al. [31] reported the presence of CD133 in both fresh human NSCLC specimens and a stabilized cell line. The authors isolated and characterized a population of CD133+ cells from NSCLC that is able to give rise to spheres that can act as tumor-initiating cells. These represent the cancer-initiating cells capable of giving rise to primary tumor growth, invasion, and distant metastatic spread.

In this study, TMAs with NSCLC specimens and IHC analysis revealed higher CD133 protein expression in NSCLC tissues than in matched tumor-adjacent tissues. This result is similar to those of studies of various malignancies that have previously found high expression of CD133 in cancer tissues [32]. However, our study shows that CD133 is also located in the nucleus. Furthermore, high CD133 expression in NSCLC correlated with certain clinical pathologic parameters, including tumor diameter, tumor differentiation, and 5-year survival rate. This is consistent with previous findings concerning high CD133 expression in the cytoplasm of NSCLC cells [33]. Kaplan-Meier analysis demonstrated that the life span of patients with negative CD133 expression was longer than that of patients with positive expression. Univariate analysis showed that CD133 expression, age, tumor diameter, and tumor differentiation were correlated with overall survival of NSCLC patients. Multivariate analysis further revealed that high CD133 expression, old age, a large tumor diameter, and a poor degree of differentiation independently predicted unfavorable overall survival of NSCLC patients.

CD133 expression has been associated with chemoresistance and increased metastatic potential in multiple human cancers, although the mechanisms underlying this remain unknown.

It is necessary to understand the various signaling pathways that are involved in the regulation as well as the maintenance of CSCs in the lung. The critical pathways involved in this present themselves as promising targets for lung cancer treatment. The signaling pathways that are involved in embryogenesis are also implicated in oncogenesis. Some of the important pathways involved in the maintenance of lung cancer stem cells include Wnt/*β*-catenin, Notch, and Hedgehog. These pathways are linked with the maintenance of tissue homeostasis and normal stem cell renewal [34]. Any deregulation of such signaling drives the activity of the CSCs in multiple cancer types, including lung cancer [35].

The Notch signaling pathway is known to regulate the differentiation of epithelial progenitors during lung development [36, 37]. Because CSCs exhibit similar properties to normal stem cells, the Notch pathway may also play an important role in lung tumorigenesis. In our research, significant association between CD133 expression and tumor differentiation was identified.

NOTCH ligands, receptors, and targets have been found in a wide range of neoplasms, including lung, breast, renal, and pancreas carcinoma, neuroblastoma, and myeloma [38–41]. In many of these tumor types, it has been shown that increased NOTCH activity promotes tumor growth, whereas NOTCH pathway blockade inhibits proliferation and/or survival. Inhibition of NOTCH signaling is therefore a promising therapeutic avenue in a wide range of cancers. Someone found that Notch signaling was expressed in both CD133+ and CD133− cells, when blocking the Notch pathway in CD133+ cells, the growth of cells was depressed, the cell cycle would be arrested in G2/M phase, and it can enhance the effect of chemotherapy [42].

Nuclear localization of CD133 may be an indicator of poor prognosis in non-small cell lung cancer, CD133 is known as a surface molecule, such anomalous localization in the nucleus has been described for several other molecules and cell-surface receptors in various malignancies [43, 44].

CD133 is a member of the pentaspan transmembrane glycoprotein family; transmembrane proteins are generally internalized by endocytosis, delivered in the cytoplasm, and then transported to the nucleus by classical pathways involving specific proteins (importins) that recognize nuclear localization signals (NLS) in cargo proteins. Macromolecules can be internalized by two major endocytic pathways, involving either caveolin or clathrin. When moving into the nucleus, CD133 can act as transcriptional regulators by interfering with molecular pathways directly connected to the proliferation and differentiation of tumor cells. But the exact mechanism is unknown and needs further study.

## 5. Conclusion

We have identified for the first time that CD133 is expressed in the cell nucleus of NSCLC tissues at a higher level than that of adjacent tissues. This increased nuclear expression was closely correlated with poor survival and as such CD133 may play an essential role as a prognostic marker of survival for patients with NSCLC while also providing a reference for clinical work. Its clinical significance is consistent with that previously described for increased cytoplasmic expression of CD133. Our study aids in understanding the roles of CD133 in the progression and development of NSCLC. Finally, related signaling pathways and the potential mechanisms of CD133 involvement in NSCLC development and progression need to be studied more intensively to further understand the prognostic and therapeutic value of CD133.

## Figures and Tables

**Table 1:** CD133 expression in cytoplasm and clinicopathological parameters in 239 NSCLC specimens.

Group	CD133 expression in nucleus				CD133 expression in cytoplasm			
	Low or no expression (%)	High expression (%)	Pearson *X* ^2^	*P* value	Low or no expression (%)	High expression (%)	Pearson *X* ^2^	*P* value
Total	102 (42.7)	137 (57.3)			114 (47.7)	125 (52.3)		

Gender								
Female	29 (49.1)	30 (50.9)	1.342	.247	27 (45.8)	32 (54.2)	0.118	.732
Male	73 (40.6)	107 (59.4)			87 (48.3)	93 (51.7)		

Age								
≤60 years	38 (43.2)	50 (56.8)	0.015	.904	41 (46.6)	47 (53.4)	0.069	.793
>60 years	64 (42.4)	87 (57.6)			73 (48.3)	78 (51.7)		

Tumor diameter								
≤4 cm	73 (48.0)	79 (52.0)	4.883	.027^*^	81 (53.3)	71 (46.7)	5.232	.022^*^
>4 cm	29 (33.3)	58 (66.7)			33 (37.9)	54 (62.1)		

Histological type								
Squamous cell carcinoma	51 (40.8)	74 (59.2)	3.296	.348	62 (49.6)	63 (50.4)	2.084	.555
Adenocarcinoma	25 (54.3)	21 (45.7)			23 (50.0)	23 (50.0)		
Adenosquamous carcinoma	24 (38.1)	39 (61.9)			28 (44.4)	35 (55.6)		
Others	2 (40.0)	3 (60.0)			1 (20.0)	4 (80.0)		

Differentiation								
Well	14 (87.5)	2 (12.5)	18.339	<.001^*^	14 (87.5)	2 (12.5)	18.479	<.001^*^
Moderate	70 (43.7)	90 (56.3)			81 (50.6)	79 (49.4)		
Poor	18 (28.6)	45 (71.4)			19 (30.2)	44 (69.8)		

Stage grouping with TNM								
Stage I	64 (52.5)	58 (47.5)	10.055	.007^*^	70 (57.4)	52 (42.6)	9.693	.008^*^
Stage II	58 (30.0)	42 (70.0)			21 (35.0)	39 (65.0)		
Stage III-IV	20 (42.7)	37 (57.3)			23 (40.4)	34 (59.6)		

^*^ *P* < .05.

**Table 2:** Univariate and multivariate analysis of prognostic factors in NSCLC for 5-year overall survival.

Variable	Years	Univariate analysis	Multivariate analysis			
		*P* value	HR	*P* value	95% CI	
CD133 expression in cytoplasm						
High versus low	5	<.001^*^	2.709	<.001^*^	1.898	3.866

CD133 expression in nuclear						
High versus low	5	<.001^*^	5.211	<.001^*^	3.525	7.703

Gender						
Male versus female	5	.655				

Age (years)						
≤60 versus >60	5	.165				

Tumor diameter (cm)						
≤4 versus >4	5	.035^*^	1.52	.005^*^	1.138	2.051

Histological type						
Sq versus Ad versus SA	5	.300				

Differentiation						
Well versus moderate versus poor	5	.001^*^	1.393	.015^*^	1.066	1.820

Stage						
I versus II versus III-IV	5	.027^*^				

^*^ *P* < .05.
